# The Impact of Lidocaine on Adipose-Derived Stem Cells in Human Adipose Tissue Harvested by Liposuction and Used for Lipotransfer

**DOI:** 10.3390/ijms21082869

**Published:** 2020-04-20

**Authors:** Felix Grambow, Rico Rutkowski, Fred Podmelle, Katrin Schmoeckel, Florian Siegerist, Grzegorz Domanski, Matthias W. Schuster, Grazyna Domanska

**Affiliations:** 1 Department of Oral and Maxillofacial Surgery/Plastic Surgery, University Medicine Greifswald, 17489 Greifswald, Germany; 2 Department of Oral and Maxillofacial Surgery, University Medical Center Hamburg Eppendorf, 20251 Hamburg, Germany; 3 Department of Immunology, University Medicine Greifswald, 17489 Greifswald, Germany; 4 Department of Anatomy and Cell Biology, University Medicine Greifswald, 17489 Greifswald, Germany; 5 Department of Neonatology and Pediatric Intensive Care, University Medicine Greifswald, 17489 Greifswald, Germany

**Keywords:** lipofilling, autologous lipotransfer, adipose-derived stem cells, lidocaine

## Abstract

The local anesthetic lidocaine, which has been used extensively during liposuction, has been reported to have cytotoxic effects and therefore would be unsuitable for use in autologous lipotransfer. We evaluated the effect of lidocaine on the distribution, number, and viability of adipose-derived stem cells (ASCs), preadipocytes, mature adipocytes, and leukocytes in the fatty and fluid portion of the lipoaspirate using antibody staining and flow cytometry analyses. Adipose tissue was harvested from 11 female patients who underwent liposuction. Abdominal subcutaneous fat tissue was infiltrated with tumescent local anesthesia, containing lidocaine on the left and lacking lidocaine on the right side of the abdomen, and harvested subsequently. Lidocaine had no influence on the relative distribution, cell number, or viability of ASCs, preadipocytes, mature adipocytes, or leukocytes in the stromal-vascular fraction. Assessing the fatty and fluid portions of the lipoaspirate, the fatty portions contained significantly more ASCs (*p* < 0.05), stem cells expressing the preadipocyte marker Pref-1 (*p* < 0.01 w/lidocaine, *p* < 0.05 w/o lidocaine), and mature adipocytes (*p* < 0.05 w/lidocaine, *p* < 0.01 w/o lidocaine) than the fluid portions. Only the fatty portion should be used for transplantation. This study found no evidence that would contraindicate the use of lidocaine in lipotransfer. Limitations of the study include the small sample size and the inclusion of only female patients.

## 1. Introduction

Autologous lipotransfer has become a standard procedure in plastic and reconstructive surgery. It has been successfully used in many areas ranging from breast reconstruction, through augmentation in facial lipoatrophy and scar correction to the adjustment of body proportions [1,2,3,4,5]. Some challenges remain to be addressed with respect to its long-term unpredictability of volume stability, e.g., in high-volume grafts and lipofilling of irradiated tissue [6,7,8]. There is still no final consent on the optimal surgical techniques to be used for lipotransfer [9,10,11,12,13,14].

Adipose tissue harvested by liposuction contains mainly mature adipocytes, erythrocytes, leukocytes, and, most importantly for high and predictable graft take, cells of the stromal-vascular fraction (SVF), including preadipocytes and adipose-derived stem cells (ASCs) [15,16,17]. ASCs’ phenotype resembles that of bone marrow-derived mesenchymal stem cells (MSCs) and can differentiate into several cell types, such as adipocytes [18,19], osteoblasts [18,20], chondrocytes [20], myocytes [18], and tenocytes [21]. These features make ASCs of high interest for use in regenerative medicine [22]. In contrast, preadipocytes are already restricted to evolve exclusively into adipocytes [23].

Lidocaine, the most commonly used and best understood local anesthetic, has been shown to have minimal systemic toxicity when used in tumescent local anesthesia (TLA) and is therefore recommended as the anesthetic agent for liposuction. A survey from the American Society for Aesthetic Plastic Surgery showed that the majority of American physicians use a tumescent solution containing 0.5 mg/mL of lidocaine [24,25,26]. 

However, in vitro trials have shown that lidocaine exerts some potentially damaging effects on ASCs, which are crucial for transplant survival [27,28]. Consequently, a potentially cytotoxic effect of lidocaine would prevent its use as local anesthetic for lipotransfer and would result in a procedure conducted under general anesthesia [29].

In this study, we focused on the effect of lidocaine, applied during TLA prior to liposuction, on cells of the SVF. Adipose-derived stem cells have been found to be crucial for transplant survival by promoting angiogenesis and decreasing cell death and therefore, were the main focus of this research [30,31]. 

## 2. Results

### 2.1. Basic Demographics and Clinical Data of the Patients

In total, 11 female patients, who presented randomly at the clinic for Maxillofacial Surgery/Plastic Surgery, Greifswald, Germany, for abdominoplasty, were included in this study. Patients’ basic demographics and clinical history are summarized in Table 1. On average, they were 42 years old (range from 19 to 60 years, standard error of the mean- SEM = 3.5), had a body-mass-index (BMI) of 30.2 kg/m^2^ (SEM = 2.06), and waist-to-hip ratio of 0.86 (SEM = 0.03). None of the patients were reported to have diabetes mellitus type 1 or 2.

### 2.2. Hematoxylin-Eosin (HE) Staining of Unprocessed Lipoaspirate and Surgically Resected Fat Tissue

The two fractions resulting from sedimentation after liposuction are termed unprocessed lipoaspirate (UPLA), which is the fatty supernatant portion, and unprocessed liposuction aspirate fluid (ULAF), which is the fluid portion of the lipoaspirate. Fat tissue was harvested by liposuction with (w/) and without (w/o) the use of lidocaine. Furthermore, in one patient, a piece of fat tissue containing lidocaine was dissected.

**Figure 1:**
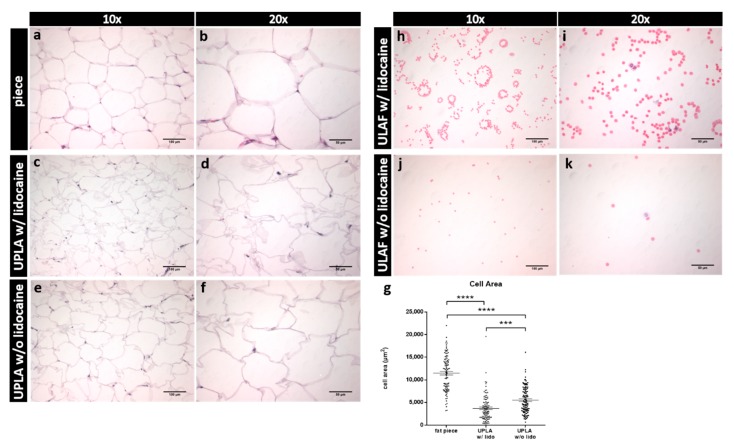
The fat piece and the unprocessed fatty portion (UPLA) after paraffin embedding and Hematoxylin-Eosin (HE) staining, as well as the unprocessed fluid portion (ULAF) after HE staining are shown. Slides were observed in a light microscope. In panels (**a**,**b**), a fat piece harvested by surgical extraction w/lidocaine is seen in 10× and 20× magnification. In panels (**c**,**d**), the UPLA harvested by liposuction w/lidocaine and in panels (**e**,**f**) w/o lidocaine is shown in 10× and 20× magnification. In panel (**g**), the cell area (in µm^2^) of the cross-sections from the fat tissue piece and the UPLA w/and w/o lidocaine of 100 random mature adipocytes (20 cells in 5 fields of views = 100 cells, 10× magnification) was compared. The mean and standard error of the mean are shown. *** *p* < 0.001; **** *p* < 0.0001. In (**h**,**i**), the ULAF of the lipoaspirate w/lidocaine, and in (**j**,**k**), w/o lidocaine is shown in 10× and 20× magnification.

A significant mechanical impact of the liposuction procedure on the integrity of mature adipocytes was observed. Cell areas of adipocytes obtained by surgical resection were significantly larger (*p* < 0.001) than UPLA samples harvested w/and w/o lidocaine (Figure 1g). Furthermore, UPLA cells of the sample w/o lidocaine were significantly larger (*p* < 0.001) than cells of the lidocaine-containing sample (Figure 1c–f). As shown in Figure 1a–g, liposuction performed with a pressure of 600 mmHg (=0.8 bar) appears to damage mature adipocytes, leading to cell shrinking.

Subsequently, the ULAF was assessed histologically (Figure 1h–k). We observed that erythrocytes were the predominant cell population in the ULAF. Additionally, some leukocytes were found in this fraction.

### 2.3. Hematoxylin-Eosin (HE) Staining, Immunostaining and Flow Cytometry of Processed Lipoaspirate

The main purpose of the research was to quantify the potential effects of lidocaine on ASCs, preadipocytes, mature adipocytes, and leukocytes number and live vs. dead status, found, after an isolation process, inside the SVF of the processed lipoaspirate. The two fractions resulting from the isolation process are termed processed lipoaspirate (PLA), which is the fatty supernatant portion, and liposuction aspirate fluid (LAF), which is the fluid portion of the lipoaspirate.

**Figure 2:**
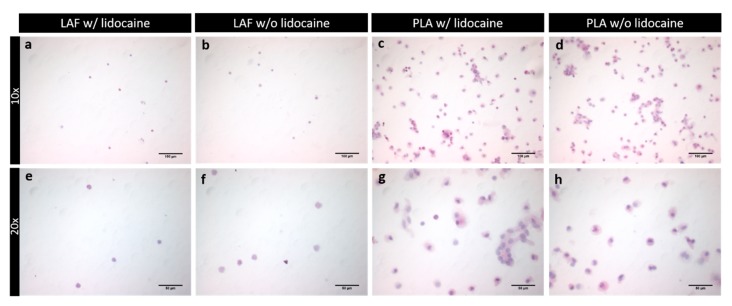
This figure presents the lysed SVF of the lipoaspirate of the fluid (LAF) and fatty portion (PLA), which was later used for flow cytometry. Slides were observed in a light microscope. In (**a**,**e**), the LAF w/lidocaine, and in (**b**,**f**), the LAF w/o lidocaine is seen in 10× and 20× magnification. In panel (**c**,**g**), PLA w/lidocaine, and in (**d**,**h**), PLA w/o lidocaine is shown in 10× and 20× magnification.

Figure 2 gives a histological illustration of the cells of the SVF. Only nucleated cells were visible, indicating the complete lysis of erythrocytes. Further differentiation and quantification of cell types was conducted by flow cytometry.

The cytotoxic effect of lidocaine was quantified by determining the relative distribution and the absolute number of nucleated cell populations of the SVF, harvested w/or w/o lidocaine. In addition, the ratio of living to dead cells was evaluated using phenotypic markers.

A significantly higher percentage of nucleated cells were found inside the PLA w/o lidocaine compared to the LAF w/o lidocaine (*p* < 0.01) in proportion to all events (cells and cell fragments) counted by flow cytometry. The PLA w/lidocaine also contained significantly more nucleated cells than the LAF w/lidocaine (*p* < 0.05). The absolute number of nucleated cells was significantly higher inside the PLA w/o lidocaine compared to the LAF w/o lidocaine (*p* < 0.05). There were no significant differences in the relative distribution and absolute number of nucleated cells between the samples w/or w/o lidocaine from the same type of isolates.

**Figure 3:**
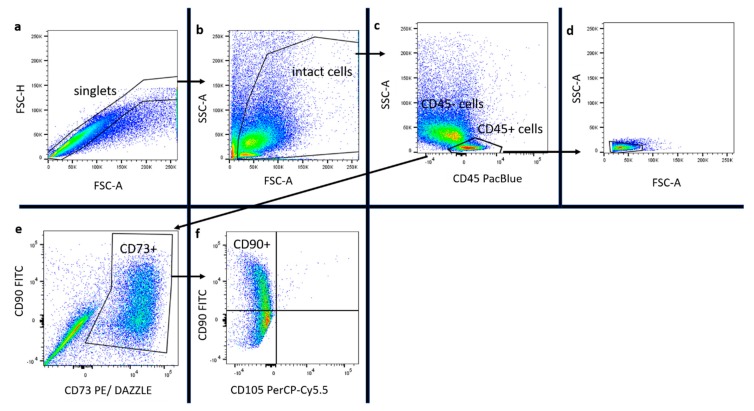
Part I of the gating strategy applied in the study, shown here with PLA cells of one patient. In (**a**), singlets were gated. In (**b**) of all singlets, intact cells were gated, leaving cell fragments aside. In (**c**) of all intact cells, CD45^+^ cells were gated. In (**d**), CD45+ cells are shown in SSC/FSC. In (**e**), the CD45- cell population was assessed for CD73 expression. In (**f**), these CD45-, CD73+ cells were assessed for CD105 and CD90 expression. Stem cells appear to be CD45-, CD73+, CD90+, and CD105-.

**Figure 4:**
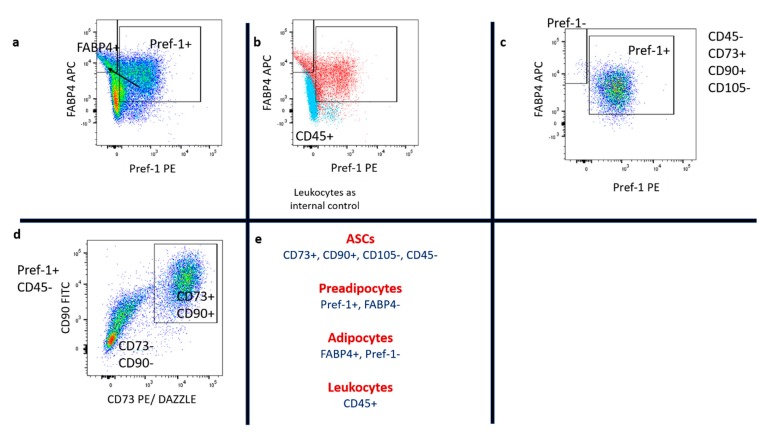
Part II of the gating strategy. In (**a**), intact cells expressing Pref-1 and FABP4 were gated. Preadipocytes lose Pref-1 expression and enrich FABP4 during further differentiation to mature adipocytes. (**b**) Preadipocytes and adipocytes were gated by using leukocytes as an internal control. (**c**) CD45-, CD73+, and CD90+ cells were assessed for Pref-1 expression. In the PLA sample, most stem cells expressed Pref-1. In (**d**), Pref-1+ and CD45- cells were assessed for CD73 and CD90 expression and found to be partly CD73+ and CD90+. (**e**) Specific marker profiles for stem cells, preadipocytes, mature adipocytes, and leukocytes are shown.

**Figure 5:**
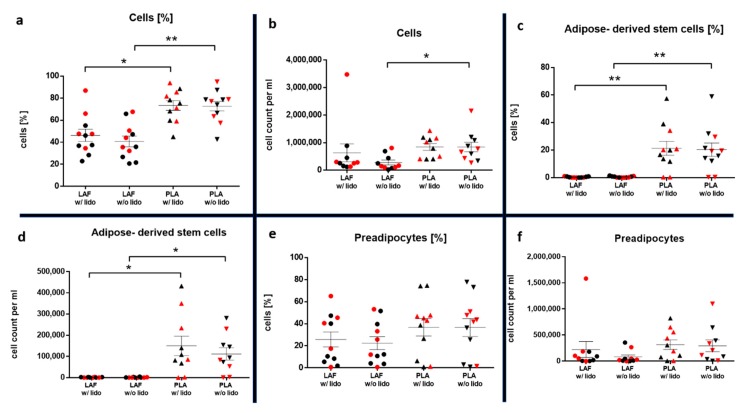
The graphs show the statistical comparison of the relative distribution (*n* = 11) and absolute number (*n* = 10) of all cells (**a**,**b**), ASCs (**c**,**d**), and preadipocytes (**e**,**f**) inside the SVF of the fluid (LAF) and fatty (PLA) portion w/ and w/o lidocaine. Samples of each patient are marked in every group. The mean and standard error of the mean in each group is shown. For multiple comparisons, the Kruskal–Wallis test was used. The α-value was at 0.05. Patients with a BMI ≥ 30 are shown in red.

The influence of lidocaine on distinct subpopulations of the SVF, such as ASCs (CD45-, CD73+, CD90+, and CD105-), preadipocytes (Pref-1+ FABP4-), mature adipocytes (Pref-1- FABP4+), and leukocytes (CD45+), was assessed as described in the methods section. The implemented gating strategy is shown in Figure 3 and Figure 4. In Figure 5a,b results of nucleated cells are shown.

The results showed a significantly higher percentage (*p* < 0.01) and absolute number (*p* < 0.05) of stem cells inside the PLA compared to the LAF, which contained almost no ASCs. No differences between the samples w/and w/o lidocaine were found (Figure 5c,d). With regard to cell viability, no differences between the two PLA treatments were detected (*n* = 5, $\bar{x}$: mean, SEM: standard error of the mean). The alive rate w/lidocaine was $\bar{x}$ = 25.5% SEM = 15.6%, and w/o lidocaine $\bar{x}$ = 28.9% SEM = 18.1%; the absolute cell number of alive cells w/lidocaine was $\bar{x}$ = 25,109/mL SEM = 15,828/mL, and w/o lidocaine $\bar{x}$ = 32,113/mL SEM = 23,670/mL (Table 2).

The preadipocyte cell yield in the PLA was not significantly higher compared to the LAF, as shown in Figure 5e,f.

No influence on the number or viability of preadipocytes inside the SVF of PLA and LAF was observed (*n* = 5, $\bar{x}$: mean, SEM: standard error of the mean). The PLA alive rate w/lidocaine was $\bar{x}$ = 70% SEM = 15%, and w/o lidocaine $\bar{x}$ = 77.9% SEM = 8.5%; the PLA absolute cell number of alive cells w/lidocaine was $\bar{x}$ = 88,756/mL SEM = 44,481/mL, w/o lidocaine $\bar{x}$ = 87,814/mL SEM = 58,406/mL; LAF alive rate: w/lidocaine $\bar{x}$ = 93% SEM = 3.4%, w/o lidocaine $\bar{x}$ = 93% SEM = 2.59%; and LAF absolute cell number of alive cells: w/lidocaine $\bar{x}$ = 38,380/mL SEM = 21,419/mL, w/o lidocaine $\bar{x}$ = 15,848/mL SEM = 7851/mL (Table 2).

**Figure 6:**
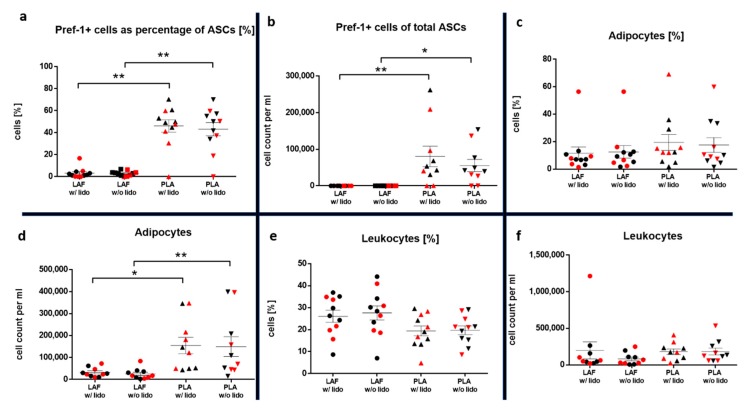
In these graphs, the relative distribution (*n* = 11) and absolute number (*n* = 10) of stem cells expressing the preadipocyte marker Pref-1 (**a**,**b**), as well as mature adipocytes (**c**,**d**) and leukocytes (**e**,**f**) inside the SVF of the fluid (LAF) and fatty (PLA) portion w/ an w/o lidocaine were compared. Samples of each patient are marked in every group. The mean and standard error of the mean in each group is shown. For multiple comparisons, the Kruskal–Wallis test was used. The α-value was at 0.05. Patients with a BMI ≥ 30 are shown in red.

Stem cells expressing the preadipocyte marker Pref-1 occurred more frequently in the relative distribution (*p* < 0.01) and absolute number (*p* < 0.01 w/lidocaine, *p* < 0.05 w/o lidocaine) in the PLA compared to the LAF (Figure 6a,b).

Mature adipocytes were found in a low number, compared to preadipocytes, in the SVF. A significantly higher absolute number of cells was found inside the PLA compared to the LAF (*p* < 0.05 w/lidocaine, *p* < 0.01 w/o lidocaine, Figure 6c,d). No influence of lidocaine on the count or viability of this type of cell was detected (*n* = 5, $\bar{x}$: mean, SEM: standard error of the mean). The PLA alive rate w/lidocaine was $\bar{x}$ = 71.2% SEM = 10.7%, w/o lidocaine $\bar{x}$ = 71.5% SEM = 11.4%; PLA absolute cell number of alive cells: w/lidocaine $\bar{x}$ = 119,035/mL SEM = 46,475/mL, w/o lidocaine $\bar{x}$ = 148,586/mL SEM = 51,609/mL; LAF alive rate: w/lidocaine $\bar{x}$ = 74.8% SEM = 8%, w/o lidocaine $\bar{x}$ = 72.5% SEM = 8.7%; and LAF absolute cell number of alive cells: w/lidocaine $\bar{x}$ = 24,944/mL SEM = 7587/mL, w/o lidocaine $\bar{x}$ = 28,163/mL SEM = 7081/mL).

Leukocytes numbers inside the LAF were, compared to the PLA, not significantly different (Figure 6e,f).

No significant difference in the viability between the four treatments was found (*n* = 5, $\bar{x}$: mean, SEM: standard error of the mean): PLA alive rate: w/lidocaine $\bar{x}$ = 80.7% SEM = 14.2%, w/o lidocaine $\bar{x}$ = 79.7% SEM = 14.4%; PLA absolute cell number of alive cells: w/lidocaine $\bar{x}$ = 124,613/mL SEM = 42,567/mL, w/o lidocaine $\bar{x}$ = 143,169/mL SEM = 55,985/mL; LAF alive rate: w/lidocaine $\bar{x}$ = 79.1% SEM = 15.4%, w/o lidocaine $\bar{x}$ = 77.8% SEM = 15.6%; and LAF absolute cell number of alive cells: w/lidocaine $\bar{x}$ = 78,334/mL SEM = 46,606/mL, w/o lidocaine $\bar{x}$ = 46,139/mL SEM = 21,961/mL).

Additional statistical analysis (one-sample t-test) of the relative distribution and absolute numbers of particular cell types (ASCs, preadipocytes, ASCs expressing preadipocyte marker Pref-1, mature adipocytes, and leukocytes) was conducted to address the sample heterogeneity. We did not observe a significant inter-patient variability (*p* > 0.05).

### 2.4. Evaluation of Lidocaine Distribution Using GC-MS

**Figure 7:**
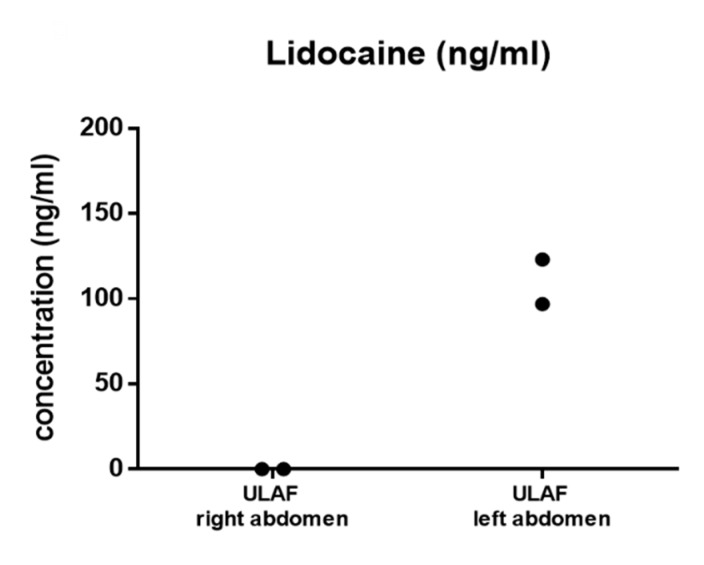
The results of the semiquantitative analysis with a GC-MS method of the unprocessed fluid portion (ULAF) of the right and left lower abdomen are shown. To evaluate the method, the lidocaine concentration was determined in 2 patients. The right abdomen, which served as a control, was free of lidocaine.

As described in the method section, lidocaine-free and lidocaine-containing TLA was injected inside the right and left side of the lower abdomen, respectively. Analysis of the fat tissue by using gas chromatography showed that lidocaine was only found inside the left abdomen. The right side of the abdomen, which served as a control, was not contaminated by lidocaine (Figure 7).

## 3. Discussion

Several studies have been carried out on the influence of lidocaine on fat tissue, delivering an inconsistent picture. Some studies described cytotoxic effects [28,32], while others did not see signs of cell damage [33,34]. The use of different methods makes it difficult to compare these results.

Research on the cytotoxicity of lidocaine was first published in 1995 by Moore et al. [35]. They postulated that lidocaine potently inhibits glucose transport and lipolysis in mature adipocytes as well as their growth in culture using in vitro experiments. Since then, the topic has been the subject of controversy. Shoshani et al. [33] and Livaoglu et al. [34] did not find effects of lidocaine on the graft take of fat tissue in animal models. In contrast, Keck et al. [28] showed a negative effect of lidocaine on ASCs’ viability after 30 min of incubation in vitro, while using a much higher concentration of lidocaine than usual for tumescence local anesthesia (2% vs. 0.062% in the therapeutic concentration). Girard et al. [32] demonstrated significant cytotoxicity of lidocaine on ASCs in vitro, using a therapeutic concentration of 0.8 mg/mL. This effect only occurred after 24 h of incubation. However, this might not reflect human in vivo conditions, because the metabolic half-life of lidocaine is about 90 min in humans [36]. In their study from 2016, Goldman et al. [37] used a method that resembles ours. They also realized liposuction w/and w/o lidocaine on symmetrical body parts, performed by the same surgeon. In contrast, they used a lower lidocaine concentration (0.3 mg/mL) and did not evaluate the presence or absence of lidocaine on each of the harvested sides. Goldman et al. [37] concluded that removing lidocaine from tumescent significantly reduced ASCs apoptosis in the lipoaspirate. Even using a concentration of 0.6 mg/mL, these findings could not be confirmed in this study.

TLA is not only used for analgesia but also for tissue inflation and vasoconstriction to reduce trauma and bleeding during liposuction. As stated, the Goldman study [37] described apoptotic effects of lidocaine on ASCs and therefore recommended the use of tumescent liposuction without lidocaine and to carry out lipotransfer under general anesthesia. They suggested infiltrating lidocaine at a later step for post-operative pain reduction. The results of our study contradict that view. Lidocaine influenced neither the relative distribution, nor absolute numbers, nor the life vs. death status of ASCs. Additionally, general anesthesia has a much higher risk in cardiorespiratory events than TLA, which has been shown to be safe up to a 35 mg/mL concentration of lidocaine [25,38]. Complications in liposuction are more common when performed under general anesthesia [39]. Furthermore, an additional TLA injection with lidocaine after liposuction would be a rather impractical approach. It would complicate the surgical procedure, not only in a logistical, but also in a time-sensitive manner.

The protocol used in our study is frequently used in the host clinic to harvest, process, and transplant/analyze harvested fat tissue and was adopted as a standard operating procedure (SOP). Potential variability in cell yields and variability in a heterogenous population (age, body mass index) was excluded through intra-individual comparison using symmetrical areas of the lower abdomen w/ and w/o lidocaine [40,41,42]. The intra-individual approach helped to eliminate fluctuations of cell yields that may occur during isolation and cell staining. Results showed no influence of a BMI ≥ 30 on cell count, which supports the validity of the results obtained by this approach. All liposuctions were performed by a single surgeon.

In addition, it was shown that when this protocol is used, lidocaine was detected only inside the LAF of the right abdomen and only cell populations from the right lower abdomen were in contact with lidocaine. This improves the reliability of the data collected.

One may argue that washing or centrifugation could, in comparison to sedimentation, decrease the contact time with lidocaine, leading to improved ASCs viability. However, several studies concluded that no processing technique is superior to another, and that centrifugation force greater than 1200× *g* causes cell damage [9]. This study used sedimentation for 25 min as an easy and accessible standard method.

To characterize cell status right before transplantation, viability has to be assessed. Cell death can be caused by either apoptosis or necrosis. Apoptosis is characterized by cell shrinking, fragmentation, and phagocytosis without inflammation. During apoptosis, phosphatidylserine is translocated from the inner to the outer leaflet of the membrane, where it can be detected by annexin V staining. In contrast, necrosis involves cell swelling, membrane perforation, and phagocytosis with inflammation [43]. Most studies that reported a cytotoxic effect of lidocaine found necrosis [32,44], whereas others claimed apoptosis [37] to be the involved mechanism. Zombie NIR™ staining, which does not differentiate between apoptosis and necrosis, was used in this study. Classic necrosis markers like propidium iodide (PI) have the disadvantage that they may leak out of cells within a short period of time [45]. Using a lidocaine concentration of 0.6 mg/mL in TLA did not show a negative influence of lidocaine on cell viability.

In contrast to this study, Goldman et al. [37] did not use a phenotypical marker profile to distinguish between different SVF cell populations. Different research groups have struggled to find a single definition of markers expressed by ASCs. Especially, the expression of the hematopoietic stem cell marker CD34 has been debated intensively [46,47]. The aim was therefore to use minimal criteria of markers to define ASCs that most research groups could relate to. The International Society of Cell Therapy (ISCT) released a statement paper in 2005 that established minimal criteria, defining bone marrow-derived MSCs as positive for CD73, CD90, and CD105 and lacking the presence of the leukocyte marker CD45 [48]. ASCs have been found to be of perivascular origin [46,49,50,51]. Zimmerlin et al. [52] evaluated the presence of MSC markers on perivascular cells and found them to express MSC markers up to 95.5%. Crisan et al. [53] also observed the expression of these surface proteins on perivascular cells. Other works questioned the expression of CD105 in freshly isolated fat tissue and suggested that it might be only expressed under culture conditions [54]. Our results show that freshly isolated ASCs do not express CD105, which is consistent with the findings of Yoshimura et al. [54]. We therefore consider CD105 a stem-cell marker in cell culture but not in freshly isolated ASCs.

Ultimately, this study defines ASCs as positive for CD73 and CD90, and negative for CD45 and CD105. Preadipocytes are already restricted to evolve into adipocytes and have been shown to express Pref-1, which acts as an inhibitor of adipogenesis and is downregulated in mature adipocytes [23,55,56,57]. This study’s findings showed that Pref-1-positive ASCs were almost entirely located inside the PLA. This leads to the conclusion that the PLA is the source of stem cells that are restricted towards an adipogenic differentiation and therefore only the fatty portion should be used for transplantation. Distinct cell populations were isolated using specific surface markers to keep the time between liposuction and data acquisition as short as possible. This approach gave the opportunity to obtain data after a short processing time, without possible variability in cell yields or viability due to cultivation of adherent cells for 24 h. The time from liposuction to transplantation usually takes from 30 min up to 2 h, depending on the surgeon and the type of surgery. Therefore, in this study, data were obtained in a similar processing time compared to a lipotransfer procedure. During further development, preadipocytes enrich FABP4, which has been used extensively as a marker for mature adipocytes [58].

Adipocytes are mitotic inactive cells that can be renewed by ASCs. In lipotransfer, they act as a filler for volume augmentation. Histological evaluation of the unprocessed lipoaspirate has shown that vibration-assisted liposuction has a negative mechanical impact on the integrity of mature adipocytes in an intra-individual approach. Cellular area was used as a surrogate parameter for cellular lysis upon liposuction. In mature adipocytes, cellular damage will cause rapid lysis, leakage of fat vacuoles, and therefore a decrease of the cell size [59]. ASCs are thought to be located inside the adipose connective tissue [46,49,50,51]. Whether the mechanical process of liposuction has a negative influence on ASC viability or a positive impact on graft survival by releasing ASCs from their original location in between adipocytes needs to be evaluated in further research.

In lipotransfer, adipocytes act as fillers for volume augmentation. It is thus important to know where to find mature adipocytes and to evaluate whether lidocaine has an impact on these cells. Adipocytes are buoyant cells. However, this study found a low number of mature adipocytes present inside the SVF even after an isolation process. Nevertheless, the main source of mature adipocytes is the upper layer of the unprocessed lipoaspirate. Transplantation of SVF cells only would therefore not fulfill the purpose of volume augmentation in lipofilling.

The influence of lidocaine on the SVF of the LAF was investigated in our study. In a previous study, Yashimuro et al. [54] found that a comparable number of cells can be harvested from the erythrocyte-lysed SVFs of PLA and LAF, with fewer LAF cells being adherent. In their study, they used plating of CD34 and CD45 expression to identify potential stem cells. Since the expression of CD34 in human adipose tissue is still debated, mesenchymal stem cell markers were used that have been shown to be expressed on perivascular stem cells in adipose tissue [46,47,50]. ASCs were located almost entirely inside the PLA.

Being a major component of the human immune system, leukocytes play a key role as a regulator in inflammation and anti-inflammation processes inside human fat tissue [60]. The effect of lidocaine on the leukocyte cell count and viability inside the SVF of PLA and LAF was therefore evaluated. Yashimuro et al. [54] showed a higher cell yield of blood-derived CD45+ cells inside the SVF of the LAF compared to the PLA. According to our study’s findings, the LAF and PLA are made up of a comparable percentage and absolute cell count of leukocytes. No impact of lidocaine was found on the viability of CD45+ cells.

It is important to emphasize that this study did not use long-term cultivation of ASCs to evaluate the effect of the potential damage done by liposuction with lidocaine on long-term survival. The conditions in cell culture do not resemble those found at the recipient site in lipotransfer due to the lack of structural, biochemical, and signaling interactions with surrounding tissues and cells [61]. Cell culture was therefore not used for this study. In vivo murine models as used by Shoshani et al. [33] are not comparable to the conditions found in humans.

To address the question of cell viability after liposuction, cell life vs. death status was assessed using Zombie NIR™ staining. Zombie NIR™ is an amine-reactive dye, which crosses the cell membranes of dead cells, thus producing information about the viability status.

The results obtained in this study give information about the cell numbers and life vs. dead status right before transplantation. The chosen study design can offer no conclusions about the long- term survival of cells in cell culture.

The two main limitations include the gender and small sample size. Only adult female patients were evaluated, which limits the generalizability of the results. There were no male patients and no children included in this study, so no conclusions can be drawn about these population groups. The small sample size prevented reasonable use of regression analysis to control for intra-individual variability. Intra-individual assessments and one-sample t-tests were applied instead, as described in the statistics section. One factor that has been described to influence the SVF cell yield is the BMI of a patient [40]. To further control for inter-patient variability, obese patients (BMI ≥ 30) were highlighted in red. Our results showed no significant inter-individual differences in cell numbers between obese and non-obese patients.

## 4. Methods

**Figure 8:**
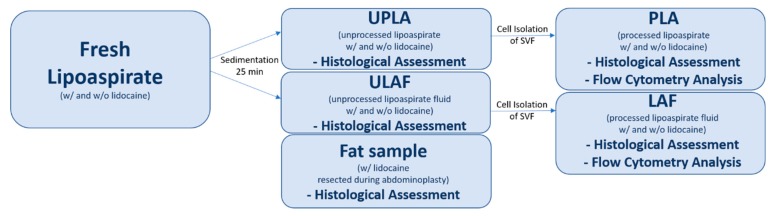
The study workflow showing the processing and evaluation of the fatty portion, unprocessed (UPLA) and processed (PLA); and the fluid portion, unprocessed (ULAF), and processed (LAF). In *n* = 1 patient, a fat piece was resected during abdominoplasty and used for histological evaluation.

Figure 8 gives an overview of the processing steps.

### 4.1. Written Informed Consent and Ethical Approval

Written informed consent was obtained from each patient. The study was carried out following the rules of the Declaration of Helsinki and was reviewed and approved by the ethics committee of the University Medicine Greifswald (Nr. BB 050/16, 05/16). All experiments were performed in accordance with local guidelines overseen by the University Medicine Greifswald and the University of Greifswald, Greifswald, Mecklenburg-Western Pomerania.

### 4.2. Tissue Harvesting by Liposuction and Abdominoplasty

**Figure 9:**
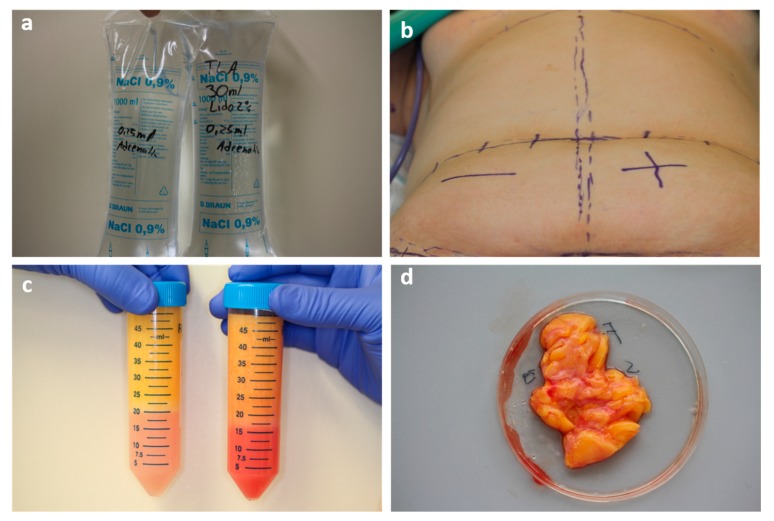
This figure shows the important steps of the harvesting method done by liposuction and abdominoplasty. In (**a**), 1l TLA with w/o lidocaine and 1l w/lidocaine (0.6 mg/mL; 0.062%) were prepared. Both contained 0.25mg of adrenalin. Panel (**b**) shows the marked abdomen just before TLA infiltration. The right lower abdomen was infiltrated w/o lidocaine and the left w/lidocaine. After liposuction, the lipoaspirate of the right (w/o) and left (w/) lower abdomen underwent 25 min of sedimentation. The unprocessed fatty portion (UPLA) was floating on the unprocessed fluid portion (ULAF) (**c**). Panel (**d**) shows the fat piece of the left (w/lido) abdomen, harvested by surgical extraction during abdominoplasty.

The right and left lower abdomen were prepared with different compositions of TLA. The right side was infiltrated with TLA containing 1 l saline (0.9%) and 0.25 mL epinephrine (1 mg/mL). The left side was prepared with 1 l saline (0.9%) containing 30 mL lidocaine 2% (600 mg) and 0.25 mL epinephrine (1 mg/mL). TLA was infiltrated under constant pressure with an 18G cannula at 20 °C temperature (Figure 9a,b).

Liposuction was performed with the vibration-assisted method, using VibraSat with 5000 cycles per minute. Rapid Extraction 9-hole cannula (diameter = 4 mm; length = 350 mm) was used under a constant negative pressure of 600 mmHg (0.8 bar). Liposuction was performed first on the right abdomen to avoid contamination of the VibraSat system with lidocaine. Lipoaspirate was collected in a falcon tube and put in an upright position for 25 min, during which the fat tissue separated by sedimentation into a fatty portion designated as unprocessed lipoaspirate (UPLA) and a fluid portion designated as unprocessed liposuction aspirate fluid (ULAF) (Figure 9c).

In one patient, lidocaine containing fat tissue was dissected as a piece by surgical extraction during abdominoplasty (Figure 9d).

### 4.3. Hematoxylin-Eosin (HE) Staining of Unprocessed Lipoaspirate and Fat Piece

In order to characterize cell damage to the fat tissue during liposuction as entirely as possible, the mechanical impact of liposuction on mature adipocytes of the UPLA was first evaluated histologically by using HE staining.

The surgically resected fat piece and the UPLA with (w/) and without (w/o) lidocaine (3 samples) were embedded in paraffin and HE stained according to the procedure described below.

Half a gram of each fat tissue sample was put in 4% paraformaldehyde over night, after which they were put through an ascending alcohol series (50%, 70%, 80%, 90%, 96%, 99%) for 2′ and two times xylol for 10′. Samples were immersed into liquid paraffin (T = 60 °C) overnight and afterwards cooled down to 20 °C. Three histological slides were cut from each sample to be stained with hematoxylin-eosin (HE). For this, the slides were put twice into xylol for 5′, a descending alcohol series (100%, 96%, 90%, 80%, 70%, 50% each 2′), and stained by putting the slides through distilled water for 2′, hematoxylin for 2′, tap water for 5′, eosin for 5′, distilled water for 1′, an ascending alcohol series (70% 1′, 80% 1′, 90% 1′, 96% 1′, 96% 1′ 100% 3′), and twice in xylol (3′ each). To quantify the damage caused by liposuction, the cell area of 100 random adipocytes from each slide (20 cells in five fields = 100 cells, 10× magnification) were measured by using ImageJ software (Research Services Branch, National Institute of Mental Health, Bethesda, MD, USA). Damaged cells appeared smaller in their surface area.

For further evaluation of the lipoaspirate, glass slides of ULAF were prepared using a cytospin technique (200× *g*, 5′). Slides were incubated in 4% paraformaldehyde for 10′ and HE stained as described above. Cells were assessed morphologically.

### 4.4. Processing of Lipoaspirate

#### 4.4.1. Cell Isolation from ULAF and UPLA

In order to isolate cells from the ULAF and the UPLA, the samples had to be processed differently, as described below. The two fractions resulting from the isolation process are termed processed lipoaspirate (PLA) and liposuction aspirate fluid (LAF).

Samples w/and w/o lidocaine, 25 mL each, were allowed to sediment for 25 min, resulting in 4 samples (ULAF w/and w/o lidocaine; UPLA w/and w/o lidocaine).

Samples of 5 mL ULAF w/and w/o lidocaine were pipetted onto a 100-μm nylon mesh of a cell strainer and centrifugated at 300× *g* for 5’. The pellets were resuspended in 500 μL phosphate-buffered saline (PBS) and centrifugated again at 500× *g* for 6´. Material remaining on the mesh and supernatants were removed after every step. Pellets containing the stroma-vascular fraction (SVF) were resuspended in 500 μL of PBS.

UPLA samples were treated with Collagenase I and Collagenase II to maximize the viability and yield of isolated cells. Then, 10 mL of Liberase TL working solution (Roche, Germany) was prepared by mixing 1 mL of Liberase TL (20 U/mL in PBS) and 9 mL of HBSS containing Ca^2+^ and Mg^2+^ (Gibco, USA). Then, 5 mL of Liberase TL working solution were added to 5 mL of UPLA w/or w/o lidocaine and incubated for 1 h at 37 °C. Samples were pipetted onto a 100-μm nylon mesh of a cell strainer, washed with 10 mL of wash buffer (HBSS with 10% FCS but without Ca^2+^ and Mg^2+^, Gibco, USA) containing DNase (20,000 IU/mL, 0.13 mL, Roche, Germany), and 5 mL of wash buffer without DNase. Samples were centrifugated at 300× *g* for 5′. Material remaining on the mesh and supernatants were removed. Pellets were resuspended in 25% Percoll (Sigma, St. Louis, MO, USA) and a density gradient centrifugation with 500× *g*, 20 min, T = 18 °C was performed. The superior liquid was removed gently, with 5 mL remaining inside the falcon tube. Cells, located at the bottom of the tube, were diluted in 20 mL of wash buffer and centrifugated at 300× *g* for 5′. Samples were than resuspended in 500 μL of PBS and centrifugated again at 500× *g* for 6′. Supernatants were removed after centrifugation. Pellets containing the SVF were finally resuspended in 500 μL of PBS.

#### 4.4.2. HE Staining of a Single Cell Suspension

Erythrocytes in the SVF suspension were selectively lysed with lysis buffer and histological slides were prepared using a cytospin technique (200× *g*, 5′). Cells were HE stained and assessed morphologically. Methods were used as described above.

#### 4.4.3. Immunostaining and Measurement by Flow Cytometry

Immunostaining was implemented to identify distinct cell populations. Surface molecular markers CD73, CD90, CD105, and CD45 were used for distinction between leukocytes and adipose-derived stem cells (ASCs). Pref-1 and FABP4 were used to identify preadipocytes and mature adipocytes. The presence of Pref-1 within the stem cell population was also evaluated. In 5 patients, Zombie NIR™ staining was included to distinguish between living and dead cells.

First, cells were washed with PBS and centrifuged for 6′ at 1200 rpm (250× *g*) at room temperature (RT). Then, 1 µL of NIR (Zombie NIR™ Fixable Viability Kit, #423106, Biolegend, San Diego, CA, USA) was added. Cells were incubated for 30′ at RT in darkness and then washed with FACS buffer (BD FACS-Flow, #342003, 2% FCS, 2mM EDTA, 0.02% NaN3, Fisher Scientific GmbH, Berlin, Germany), centrifuged for 6′ at 1200 rpm (250× *g*) at RT. Then, 1 µL of Fc-Block (#130-092-575, Miltenyi Biotec GmbH, Germany) was added and the mixture was incubated for 5′ at 4 °C. Afterwards, 50 µL of primary antibody mix: Pref-1 (PE, rat IgG1 clone 24-11, #D187-5, MBL International, Woburn, MA, USA), CD73 (PE/Dazzle, Mouse IgG1, κ Clone AD2, #344019, Biolegend, USA), CD90 (FITC, Mouse IgG1, κ Clone 5E10, #328107, Biolegend, USA), CD105 (PerCP-Cy5.5, Mouse IgG1, κ Clone 43A3, #323215, Biolegend, USA), CD45 (PacBlue, Mouse IgG1, κ Clone HI30, #304021, Biolegend, USA) was added and cells were incubated for 30′ at 4 °C. In CD73, CD90, and CD105, FMO was used. For Pref-1 staining, isotype control (PE, rat IgG1, clone 1H5, #M080-5, MBL International, USA) was used. To lyse erythrocytes, 4 mL of lysis buffer (#555899, BD Pharm Lyse, BD, USA) was added and incubated for 7′ at RT in darkness. The mixture was centrifuged for 6′ at 1200 rpm (250× *g*), washed again with FACS buffer, and centrifuged for 6′ at 1200 rpm (250× *g*). Then, 100 µL of solution A Fix & Perm (#GAS-002, ADG, Vienna, Austria) were added for 15′ at RT in darkness and cells were washed with Saponin-FACS-buffer (FACS buffer with 0.1 % Saponin, Merck, Germany), and centrifuged again for 6′ at 1200 rpm (250× *g*) at RT. Then, 100 µL of solution B with intracellular antibody FABP4 (#13063r, polyclonal rabbit IgG, Bioss Antibodies, Woburn, MA, USA) were added for 15′ at RT in darkness. Cells were washed with Saponin-FACS-buffer and centrifuged for 6′ at 1200 rpm (250× *g*). Subsequently, 100 µL of solution B with secondary antibody (APC, goat F(ab’)2, #31984, Life Technologies, Carlsbad, CA, USA) were added for 15′ at RT in darkness. Finally, cells were washed with Saponin FACS buffer, centrifuged for 6′ at 1200 rpm (250× *g*), resuspended in 100 µL of FACS buffer, and measured.

In the gating strategy, first, singlets were gated and intact cells detected, leaving cell fragments aside. Intact cells were assessed for CD45 expression, and the CD45- population was evaluated for the expression of CD73, CD90, and CD105. ASCs were found to express the pattern CD45-, CD73+, CD90+, and CD105-. ASCs were stained with Zombie NIR™ to assess their viability. Zombie+ cells were counted as non-viable.

Preadipocytes and mature adipocytes were identified by using Pref-1 and FABP4 staining. Preadipocytes showed high Pref-1 expression and enriched FABP4 during further differentiation to mature adipocytes. Preadipocytes (Pref-1+ FABP4-) and adipocytes (Pref-1- FABP4+) were gated by using leukocytes (CD45+) as an internal control.

Subsequently, the co-expression of stem cell markers (CD45, CD73, CD90, and CD105) with preadipocyte and adipocyte markers (Pref-1 and FABP4) was evaluated.

For this purpose, CD45-, CD73+, CD90+, and CD105- cells were assessed for Pref-1 and FABP4 co-expression. Preadipocytes, leukocytes, and adipocytes were assessed for viability by Zombie NIR™ staining. Zombie+ cells were counted as non-viable. Compensation for individual molecular markers was done with single stained fixed cells (NIR and FABP4) and beads (Pref-1, CD45, CD73, CD90, CD105, BD, USA). The implemented gating strategy is shown in Figure 3 and Figure 4.

The absolute number of SVF cells was counted using BD Trucount™ Tubes (BD Biosciences, USA). For this, a known number of beads was added to the samples. Beads were counted by flow cytometry and the absolute cell numbers were therefore calculated. Erythrocytes were than selectively lysed, using lysis buffer, and the absolute number of remaining cells per mL was determined again using BD Trucount™ Tubes. The method differentiated between cell fragments and intact cells using flow cytometry.

Cell populations of PLA and LAF samples w/and w/o lidocaine were expressed either on a percentage basis (*n* = 11) or in absolute numbers (*n* = 10). The number of measured patients differed due to technical issues using the BD Trucount™ tubes. Therefore, the absolute cell number could not be determined in the first patient. Viability was only determined in the last 5 patients. The mean ($\bar{x}$) and standard error of the mean (SEM) of alive ASCs, preadipocytes, mature adipocytes, and leukocytes were calculated in absolute cell numbers/mL and as a percentage of all counted cells of each cell type found inside the PLA and LAF w/and w/o lidocaine.

### 4.5. GC-MS Evaluation of Method

During the surgery, lidocaine could have diffused into the lidocaine-free right side of the lower abdomen. Lidocaine concentration was determined inside the LAF of the right and left lower abdomen to prove the method’s reliability.

Lidocaine concentration was determined in the LAF samples of 2 patients, harvested from the right and left abdomen, using a GC-MS method after liquid-liquid extraction from the organic matrix.

The extraction of lidocaine from the matrix was conducted according to the following method. First, 1 mL of each native sample was extracted with 4 mL of methyl-tert-butyl ether (MTBE) by shaking for 5 min followed by centrifugation (4000× *g*; 10 min) at room temperature. The upper organic layer was collected and evaporated to dryness under a gentle air stream at 40 °C. Afterwards, the residues were dissolved in 200 µL of methanol, and an aliquot of 1 µL of each sample was injected into the chromatographic system.

The measurements were performed with a gas chromatographic system (Hewlett Packard, 5890 series II, Alexandria, VA USA) equipped with a mass spectrometric detector (Hewlett Packard, 5972 series, USA). The column used was HP5MS (30 m × 0.25 mm × 0.25 µm). One run required 18 min over a temperature program (100–300 °C). The MS system worked in the single ion mode (SIM), and the interpretation of the signals happened over the peak areas.

### 4.6. Statistics

Data were tested for normality using a D’Agostino omnibus K^2^ test. Comparisons between multiple variables were done using the Kruskal–Wallis test. The differences between groups were accepted to be significant if α-value was smaller than 0.05.

We used the following procedure to test the sample heterogeneity. First, the difference between the relative cell distribution and absolute cell numbers between groups w/and w/o lidocaine were calculated and tested for normality using D’Agostino omnibus K^2^. Then, a one-sample t-test was applied on the normally distributed results. The theoretical mean was set to 0. Lidocaine was declared to have no influence on the cell distribution and number if the α-value was higher than 0.05. Statistical tests were performed with Prism GraphPad 6 software (Research Services Branch, National Institute of Mental Health, Bethesda, MD, USA).

## 5. Conclusions

In conclusion, lidocaine has no negative impact on the distribution, cell number, and viability of ASCs and preadipocytes. Only the fatty portion (PLA) contains ASCs, which are crucial for successful lipotransfer. Pref-1+ stem cells, which are restricted to adipogenic evolvement, were found to be significantly more common inside the PLA than inside the LAF (*p* < 0.01). Mature adipocytes, which are needed for volume augmentation, were almost entirely found inside the UPLA.

## Figures and Tables

**Table 1:** In Table 1, the demographics (age, body-mass-index (BMI), waist/hip ration) and clinical history (weight gain/loss, diabetes) are shown for each patient.

Patient	Age	BMI	Weight Gain/Loss in Last 6 Months	W:H Ratio	Diabetes
1	43	26	no	0.854	no
2	44	35	yes, −5 kg	1.026	no
3	35	48	no	0.91	no
4	30	30	yes, −5 kg	0.63	no
5	41	26	yes, −5 kg	0.78	no
6	27	26	yes, −5 kg	0.85	no
7	48	29	no	0.85	no
8	37	25	no	0.97	no
9	52	34	no	0.96	no
10	60	25	yes, −5 kg	0.83	no
11	19	32	no	0.8	no
Mean	42	30.2		0.86	
SEM	3.54	2.06		0.03	

**Table 2:** Mean ($\bar{x}$) and standard error of the mean (SEM) of alive ASCs and alive preadipocytes (Zombie-) in the absolute number per mL and as a percentage of all counted stem cells and preadipocytes (*n* = 5 patients).

				w/Lido	w/o Lido
ASCs (viable)	PLA	%	$\bar{X}$	25.5	28.9
			SEM	15.6	18.1
		Cells/mL	$\bar{X}$	25,109	32,113
			SEM	15,828	23,670
Preadipocyte (viable)	LAF	%	$\bar{X}$	93	93
			SEM	3.4	2.59
		Cells/mL	$\bar{X}$	38,380	15,848
			SEM	21,419	7851
	PLA	%	$\bar{X}$	70	77.9
			SEM	15	8.5
		Cells/mL	$\bar{X}$	88,756	87,814
			SEM	44,411	58,406

## Abbreviations

ULAF	Unprocessed liposuction aspirate fluid
UPLA	Unprocessed lipoaspirate
LAF	Processed liposuction aspirate fluid
PLA	Processed lipoaspirate
MSC	Mesenchymal stem cells
ASC	Adipose derived stem cells
SVF	Stromal-vascular fraction
SEM	Standard error of the mean
GC-MS	Gas chromatography mass spectroscopy
